# Incentivizing Hospital Quality Through Care Bundling

**DOI:** 10.1002/hec.70024

**Published:** 2025-08-17

**Authors:** Katja Grašič, Adrián Villaseñor, James Gaughan, Nils Gutacker, Luigi Siciliani

**Affiliations:** ^1^ Centre for Health Economics University of York York UK; ^2^ Department of Economics and Related Studies University of York York UK

**Keywords:** incentives, pay for performance, provider behavior, quality

## Abstract

Policymakers increasingly implement pay‐for‐performance schemes to incentivize quality of care. A key design issue when incentivizing several process measures of quality relates to whether the payment should be linked to the performance on each measure or whether the payment should be conditional on all of the process measures of quality being provided, which we refer to as “care bundling”. After developing a theoretical framework of provider incentives under care bundling, we employ a difference‐in‐difference analysis to evaluate the Best Practice Tariff for fragility hip fracture, introduced in England in 2010, which rewards providers based on a care bundle of nine process measures that need to be jointly achieved. The design of the processes was evidence‐based and the size of the bonus was significant, up to 20% of the baseline tariff. The results suggest that the policy was successful in increasing the proportion of patients for whom all of the criteria are met by 52.5 percentage points in the first 5 years after its introduction. Temporal ordering of processes might matter under care bundling, but we do not find evidence that English providers exerted less effort to meet process measures if they already failed to meet an earlier one. Overall, we find that a scheme based on care bundle, which is evidence based and uses a sizable bonus, can be effective in improving hospital performance.

## 1 Introduction

Policymakers are increasingly implementing pay‐for‐performance (P4P) schemes to incentivize the adoption of best practice, thereby reducing unwarranted practice variation and improving the overall quality and efficiency of the healthcare system.^1^ Despite the international movement toward P4P schemes, the evidence about their effectiveness remains inconclusive with some studies reporting substantial improvements in quality whereas others fail to identify them (Milstein and Schreyoegg 2016; Markovitz and Ryan 2017; Zaresani and Scott 2021). Design features of the P4P schemes, such as the size of the financial incentives or the modalities by which payments are determined, may drive some of this heterogeneity (Mendelson et al. 2017; Emmert et al. 2012; Van Herck et al. 2010).

Most P4P schemes incentivize improvements in process measures of quality, while others incentivize health outcomes (Milstein and Schreyoegg 2016). A key design issue in P4P schemes with multiple incentivized performance measures is whether separate bonus payments should be made for each measure provided, or whether a single bonus payment should be made conditional on all measures being provided jointly, which we refer to as “care bundling”. Conditioning payment on a bundle of processes can give strong incentives to providers to deliver them all, but may discourage some providers to deliver any at all if they find at least one of the processes to be particularly costly.

In this study, we contribute to the literature on P4P and its design features by analyzing the effectiveness of a national P4P scheme that incentivizes hospitals through a single additional payment for every patient that receives a care bundle of nine process measures. The Best Practice Tariff (BPT) for fragility hip fracture, introduced in the English NHS in 2010, seeks to ensure timely access to surgery, involvement of geriatricians throughout the entire care pathway, and tertiary prevention of fractures. These process measures reflect best practice standards developed by the British Geriatric Society and the British Orthopedic Association (2007) on the basis of clinical evidence and professional consensus. BPT payments are made for each patient conditional on the delivery of the *entire* care bundle, that is hospitals do not receive the bonus payment for patients for whom one or more process measures are not achieved. A second distinctive feature of this P4P scheme is that the size of the financial incentive is economically significant and amounts to up to 20% of the baseline episodic payment to hospitals. This is important because one of the reasons for the lack of provider response is the relatively small payment (typically around 5% of the revenues (Cashin et al. 2014)).

After providing a theoretical framework of provider incentives under care bundling, we implement a difference‐in‐difference (DID) strategy to identify the causal effect of the BPT policy on care provision in England using Wales as a control group. Both countries have similar healthcare systems and share key institutional features such as training and regulation of healthcare professionals, free care at the point of use, and population demographics. Furthermore, hospitals in both countries report to the same clinical audit, the National Hip Fracture Database (NHFD), which ensures that achievements of the incentivized care standards are recorded and disseminated to the public in a consistent way.

Our results suggest that a P4P scheme based on care bundling, which is evidence based and awards a sizable bonus payment, is effective in improving hospital performance to a large extent. Specifically, we find that the BPT increases the proportion of patients who receive the complete care bundle by 52.5% points (pp). There is considerable heterogeneity in the impact of the BPT on the set of process measures that are incentivized with the largest improvements occurring in the involvement of geriatricians in the care process (between 17 and 56 pp) and much smaller effects in others, for example falls prevention (4.5 pp). The size of the improvement across process measures is inversely related to pre‐policy achievement levels as English hospitals seek to establish a similar level of achievement across all process measures to maximize revenues. We do not find evidence that English providers continue to exert efforts to deliver targeted process measures once they have failed to meet at least one measure, that is when the financial incentive is removed.

Our analysis makes several contributions to the literature and offers new insights into the optimal design of P4P schemes and the behavioral response of providers. First, it provides evidence on the use of a P4P payment rule which incentivizes a care bundle to incentivize quality of care. Our empirical results show that the care bundle payment can be effective in stimulating provider effort. Unlike the more common P4P arrangements with a separate payment for each of the incentivized measures, care bundles have only one payment which is conditional on satisfying all the incentivized measures in an all‐or‐nothing approach. This creates a distinct decision problem for the provider, who needs to balance the cost of delivering all the measures against the prospect of a single payment. We characterize these incentive issues in the theoretical model in Section 4. The key insight is that for some providers the bundled price will increase the scope of providing care to patients as this is the only way to gain the payment. Instead, other providers with a relatively high cost of one of the process measures of quality may not respond at all under care bundling, while they would have partially responded under a scheme incentivizing each individual process. The comparison again highlights the financial incentive given by the bundled payment to provide *all* or *nothing*. We also show that this insight holds even in the presence of synergies on costs across processes and that, as intuitively expected, the presence of cost synergies increases the scope of providing bundled care, for a given incentive scheme. We also briefly show that the case for a bundled payment is reinforced by the presence of synergies on health benefits across care processes.

Second, our study provides new evidence on the marginal contribution of P4P over and above other common policy leavers, such as the dissemination of clinical guidelines, increased monitoring of care processed, and public reporting of comparative performance information. Existing P4P schemes have often been implemented alongside other policy leavers thereby making it difficult to isolate the effect of financial incentives. For example, in order to operationalize the UK Quality Outcome Framework, the largest and most widely studied P4P scheme in primary care internationally, family doctors were given new quality standards and were required to improve their data recording and monitoring systems. Data on the quality of care of each practice were reported to the payer on a regular basis and were published in the public domain to inform patient choices. In contrast, the fragility hip fracture BPT draws on an existing data collection and incentivizes care standards that had been agreed upon previously and where provider performance was already reported publicly. Hence, both the treatment (England) and the control (Wales) group in our study experience the same non‐financial stimuli. This increases our confidence that any difference in post‐policy behaviors can be attributed to the BPT policy.

Third, our study is one of few to evaluate a high‐powered quality improvement scheme with considerable bonus payments of up to 20% of baseline payments. As mentioned above, the lack of response to previous P4P schemes may have been due to the small size of the financial bonuses (Milstein and Schreyoegg 2016), which historically have not exceeded 5% of the base price (Cashin et al. 2014) and, thus, may be insufficient to compensate providers for their additional costs. A more recent review indicates some larger bonus sizes. However these schemes are generally outside the hospital setting and remain below 10% in OECD countries where bonus size is reported Zaresani and Scott 2021. The size of the BPT bonus increased throughout the study period from 7% to 20% of the baseline payment, which permits us to test empirically how the size of financial incentives affects provider behavior.

Fourth, we contribute to a sparse literature on the effect of P4P in the clinical area of hip fracture care. Fragility hip fractures are common in elderly people and are a lead cause of mortality and morbidity, with associated disability, need for long‐term institutional care, and high medical costs (Tajeu et al. 2014). In 2000, an estimated 1.6 million hip fractures occurred worldwide, and this number is expected to increase to 6.3 million by 2050 (Cooper et al. 2011). While P4P has been implemented in various settings covering a range of conditions, we are not aware of other P4P schemes for hip fracture outside of the English NHS, despite its high health burden. A previous study by Metcalfe et al. (2019) found that the introduction of the hip fracture BPT in the English NHS led to a 1.7% point reduction in 30‐day mortality compared to a control group of patients treated in Scotland. Importantly, Metcalfe et al. (2019) did not examine how the BPT policy affects hospital achievements of incentivized process measures, a prerequisite for a causal effect on mortality,^2^ nor how the care bundle approach affects hospital decision‐making. We extend previous work in these directions.

The study is organized as follows. Section 2 reviews the related literature. Section 3 discusses the institutional setting in which providers operate. Section 4 outlines a theory model of bundled payment arrangements and implications for provider behavior. Sections 5 and 6 describe the data and the details of the empirical approach. Section 7 presents the empirical findings. Section 8 is devoted to discussion and concluding remarks.

## 2 Related Literature

Our research contributes to the literature within the broader area of hospital incentive schemes. Milstein and Schreyoegg (2016) reviewed P4P programs covering the inpatient setting across OECD countries and found that, out of 34 programs in the sample, approximately half lacked any statistical evaluation. The existing evaluations often experienced design issues, including lack of a suitable control group, which may lead to potential bias. While the review uncovered large heterogeneity across the programmes in respect of incentive design and clinical areas covered, they were all typically associated with small size of the P4P bonus and generated only limited improvement in performance. Additional P4P reviews confirm the modest effect of P4P schemes on changing providers' behavior (Mendelson et al. 2017; Eijkenaar et al. 2013a, 2013b; Mokhtary et al. 2024; Zaresani and Scott 2021; Mathes et al. 2019). A recent systematic review of systematic reviews confirms that findings from studies of P4P schemes remain mixed (Wagenschieber and Blunck 2024).

Existing reviews also commonly suggest that the reason for the limited success of the financial incentives lies in the particular design features (Eijkenaar et al. 2013a, 2013b; Ogundeji et al. 2016; Milstein and Schreyoegg 2016; Scott et al. 2016). A meta‐analysis of P4P effects estimates shows that P4P schemes incentivizing process measures generate larger responses than those incentivizing health outcomes measures (Ogundeji et al. 2016). Eijkenaar et al. (2013a, 2013b) compared different remuneration methods, including separate payment for each P4P incentive and the *“all‐or‐nothing”* arrangement where providers receive bonus payments only once a certain threshold across patients is met.^3^ The study finds advantages and limitations of different payment arrangements, noting that the optimal financial bonus structure depends on the specific incentivized program. Milstein and Schreyoegg (2016) found that the payment based on absolute scores is usually preferred over the relative rankings, while Scott et al. (2016) and Zaresani and Scott (2021) found that schemes which base reward on improvements over time have lower probability of being effective compared to those that reward performance at a single time point. Zaresani and Scott (2021) also found weak evidence of an association between incentive size and scheme effectiveness.

In contrast to the existing literature, the care bundle arrangement used in the BPT for hip fracture in this study, by which a provider must meet several criteria on a per‐patient basis to receive the bonus, does not typically feature in the P4P payment design. This is despite evidence‐based care bundles being often considered to deliver best clinical care and hence promoted as best practice. A study of very‐low birth‐weight babies from 32 neonatal departments in Germany found that an intervention bundle is feasible and can reduce bloodstream infections in neonatal departments (Salm et al. 2016). López‐Cortés et al. (2013) found that evidence‐based bundle of six adjunctive measures improved the management of patients with bacterial blood infections and reduced mortality. Similar results were found by Takesue et al. (2015). Care bundles were further shown to be effective in the ICU to reduce ventilator‐associated pneumonia and improve outcomes (Resar et al. 2005). Our study shows that P4P can be an effective tool for policymakers to promote adherence to evidence‐based care bundles, thereby improving care processes and, subsequently, patient outcomes.

More specifically, our analysis extends and improves previous studies evaluating the BPT for hip fracture. The initial assessment of the BPT hip fracture scheme (McDonald et al. 2012) suggested a positive effect of the policy on the uptake of four criteria that were included in the study. The analysis was based on aggregated hospital level data covering 1 year pre‐ and post‐policy. Our study extends this study by using a longer pre‐ and post‐policy period and providing causal estimates of the effect of the BPT policy, as well as exploring heterogeneous effects and potential mechanisms at work. While the evidence of the effect of the hip fracture BPT on adherence is limited, there is some research on its effect on patients outcomes, measured by mortality. Metcalfe et al. (2019) compared the outcomes of patients in England to those in Scotland (which is not part of NHFD) and found a 1.7% point reduction in 30‐day mortality between 2010 and 2016 as a result of the BPT in England. Similarly, Neuburger et al. (2015) identified a statistically significant fall in 30‐day mortality in England of 1.8% one year after the introduction of the BPT.

## 3 Institutional Background

Public healthcare in the UK is funded through general taxation and is free to patients at the point of use. The delivery of healthcare is decentralized, with each of the four countries of the UK (England, Scotland, Wales, and Northern Ireland) operating their own National Health Service (NHS) to provide primary and secondary care services to their resident populations. From the founding of the UK public healthcare system in 1948 until the political devolution in 1999, England and Wales operated a common NHS with shared resources and policies. While priorities and policies in both countries have begun to diverge since then, the organization of the health services still remains broadly comparable to this date. For example, both systems have similar healthcare expenditures per capita^4^ and they continue to share the same professional regulation (e.g. on clinical training, conduct, and fitness to practice) and similar pay structure for their doctors and nurses (OECD 2016). Care pathways for hip fracture patients are also similar, with patients in both countries accessing emergency care either by presenting at a hospital emergency department (ED) (e.g. arriving by ambulance, self‐referral) or by urgent referral from their family doctor. Clinical guidelines for hip fracture care are issued by the National Institute for Health and Care Excellence (NICE) and apply to both countries equally.^5^

One important aspect in which the English and Welsh NHS differ is in how they reimburse hospital providers for the care they deliver. Welsh hospitals are paid via a capitation system, where each hospital receives a lump sum that is linked to the size of local population they serve; not to the actual volume or quality of service provided. Reimbursement for the hip fracture patients is included in this sum and there is no further bonus paid to hospitals that meet best practice standards. Conversely, hospitals in England are reimbursed via a prospective payment system that was introduced in 2003 and now covers more than 60% of total hospital activity (Grasic et al. 2013). Patients are categorized into distinct healthcare resource groups (HRGs; similar to DRGs in other countries) according to their age, severity and the care that was provided. Hip fracture patients fall mostly into one of 10 orthopedic HRGs, typically related to major hip procedures, although patients may also be grouped to other HRGs if there are significant concomitant medical conditions (e.g. a stroke).

**FIGURE 1:**
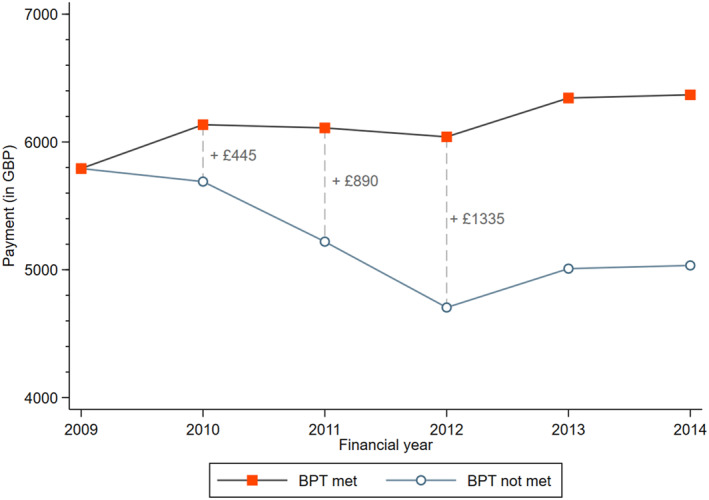
Reimbursement when BPT is met and baseline payments.

Until March 2010, English hospitals were paid a base price for each hip fracture patient, where the price reflected the historical average costs of treating patients in this particular HRG. In April 2010, the BPT for fragility hip fracture was introduced to incentivize hospitals to deliver best practice care processes according to the definitions set out by the relevant medical societies (Department of Health 2010). Under this system, hospitals now receive a lower base price $\left(P_{0}\right)$ for all patients, irrespective of the quality of care provided. In addition, they can earn a relatively large bonus payment $\left(P_{b}\right)$ on top of this base payment for each patient for whom the full set of BPT criteria are met. In the financial year^6^ 2010/11, this bonus payment amounted to £445, which was subsequently increased to £890 in 2011/12 and to £1335 in 2012/13^7^ (see Figure 1).

During our study period,^8^ hospitals received $P_{0}+P_{b}$ for each patient for whom all of the following criteria were met, and $P_{0}$ otherwise:

- BPT1: Surgery within 36 h;
- BPT2: Shared care by surgeon and geriatrician;
- BPT3: Care protocol agreed by geriatrician, surgeon and anesthetist;
- BPT4: Pre‐operative cognitive function assessment (introduced in 2012);
- BPT5: Post‐operative cognitive function assessment (introduced in 2012);
- BPT6: Perioperative assessment by geriatrician;
- BPT7: Geriatrician‐led multidisciplinary rehabilitation;
- BPT8: Secondary prevention including falls;
- BPT9: Bone health assessment.

In line with these BPT criteria, patients should be operated on within 36h from the time they present at the ED or—if the patient was not admitted via the ED—the time of diagnosis. This reflects empirical evidence that timely surgery can improve survival, decrease length of stay and the incidence of pressure ulcers, and facilitate a return to independent living (Lee and Elfar 2014). The BPT also greatly emphasizes the role of ortho‐geriatricians in the treatment of hip fracture patients, with four of the nine criteria requiring their direct involvement (BPT2, BPT3, BPT6, BPT7). Geriatricians are expected to see each patient in the perioperative period, that is within 72h of admission (BPT6), to ensure their fitness for surgery. They also should coordinate with the orthopedic surgeon and agree on the type of care the patient should receive (BPT2). Furthermore, they should be involved in the development of care protocol for patients with hip fracture (BPT3) and coordinate their activities with the rehabilitation team (BPT7), which has been shown to reduce length of inpatient stay (Cameron 2005; Kalmet et al. 2016; Lau et al. 2017). The latter two criteria are not necessarily achieved for each patient separately, but serve as a general set of rules for patients treated for hip fracture. Since April 2012, all patients must also receive a simple “memory test” type of assessment at two time points (BPT4, BPT5). Finally, patients with hip fracture are at increased risk of falls, and the BPT therefore incentivizes preventive activities such as medication review, physiotherapy work to improve strength and balance, and an assessment of the home environment (BPT8). Because many hip fracture patients have osteoporosis, they should undergo bone strengthening treatment and/or bone density scans (BPT9) to reduce the risk of future fractures.

These criteria follow closely national clinical standards for hip fracture care set by the British Orthopedic Association and British Geriatrics Society and monitored through a collaborative clinician‐led audit, the National Hip Fracture Database (NHFD),^9^ which was launched in April 2007 (Neuburger et al. 2015). The aim of the audit is to comprehensively describe the quality of care delivered to fragility hip fracture patients in the four UK countries, and to facilitate benchmarking among hospital providers and health care systems (British Orthopaedic Association 2007). Hospital managers and clinicians in participating hospitals benefit from regular regional and national meetings to share information and receive peer support (NHFD 2010). Performance data has been published in annual reports since 2009 (covering the period from October 2007 to September 2008), creating non‐financial incentives to improve in both countries. The hip fracture BPT relies on the data from the NHFD to assess compliance against the incentivized criteria.

## 4 Theoretical Framework

To fix ideas, we provide a simple model of provider behavior under a payment which rewards bundled care, and then compare to a system where the payment is not bundled. The aim of the model is to gain insights into provider incentives given by a bundled payment, as we observe for the hip fracture P4P scheme in our study, relative to one that incentivizes each process separately, as it was the case for example for stroke, another emergency condition, in England (Kristensen et al. 2016). As perhaps intuitively expected, the model shows that providers are more likely to provide bundled care under a bundled payment. Depending on the relative cost of each process faced by providers, there are scenarios where a provider would provide both processes or no processes under bundled care, but would instead provide only one process when each is financially incentivized separately. Our model contributes to the health economics theoretical literature on P4P, which has focused on multi‐tasking (Eggleston 2005; Kaarboe and Siciliani 2011; Mak 2018), gaming (Kuhn and Siciliani 2009) and selection effects (Lisi et al. 2020), and more broadly to the literature on provider incentives (Ellis and McGuire 1986; Ma 1994; Chalkley and Malcomson 1998). However, none of these studies focuses on bundling.

Consider a hospital treating an emergency patient. The hospital can treat the patient with some basic care, or can provide the patient with two additional care processes, 1 and 2, that generate additional patient benefits (and costs for the provider). For simplicity, we assume that all the patients are the same and do not differ in severity. The hospital has four different treatment options: (i) a basic treatment, (ii) the provision of basic treatment and care process 1, but not care process 2; (iii) the provision of basic treatment and care process 2, but not care process 1; and (iv) the provision of basic treatment and care processes 1 and 2.

The cost of process 1 and 2 is respectively defined with $c_{1}$ and $c_{2}$. If both care processes are provided, the hospital sustains costs $c_{12}$. We allow for the possibility of cost synergies so that $c_{12}=c_{1}+c_{2}-s$, where s≥0 measures the extent of the cost synergies. Similarly, the patient benefit from process 1 and 2 is respectively defined with $b_{1}$ and $b_{2}$, and $b_{12}$ if the processes are jointly provided. We assume that there are no fixed costs in our model, and that treating each patient generates a strictly positive marginal cost. This can be thought of as an approximation. In practice, doctors are salaried. However, expanding the provision of one service is still likely to have financial implications. To provide a new service to a large group of patients, additional doctors may have to be recruited. Alternatively, some time of the doctors could be freed by recruiting additional nurses, which will also be costly, therefore shifting some tasks from doctors to nurses. Another possibility is that doctors will have to reduce services in other domains, which can also lead to financial losses for other DRGs.

We assume that providers maximize their financial surplus, defined with $\pi_{i}$, where i=1,2,12 denoting the cases when care processes 1, 2 or both are provided. We further assume that providers differ in costs, for example to reflect different degrees of efficiency in providing processes 1 and 2, so that $c_{1}$ and $c_{2}$ are distributed with joint density function $f\left(c_{1},c_{2}\right)$ over the support $c_{1}\in \left[\left.k_{1},+\infty \right)\right.$, $c_{2}\in \left[k_{2},+\infty \right)$.^10^

**FIGURE 2:**
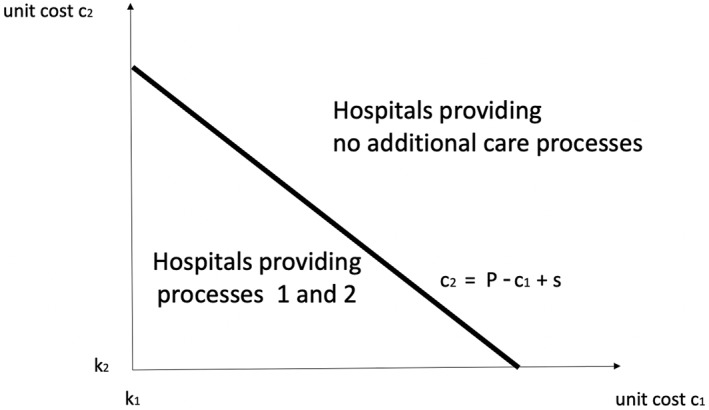
Bundled payment.

Under bundled payment, we assume that a funder pays a price P if both care processes are provided, and zero otherwise (and this price is above the sum of minimum provider costs, $P>k_{1}+k_{2}$). Under these arrangements, the provider has an incentive to provide both processes if $\pi_{12}>0$, or, more explicitly, if $P>c_{1}+c_{2}-s$. The solution is described in Figure 2. It shows that only providers with relatively lower costs on care processes 1 and 2 have an incentive to provide them. Providers with relatively high costs provide neither processes. It is also immediate to see that higher cost synergies or a higher bundled payment will induce more hospitals to provide both care processes (the diagonal line shifts upwards).

**FIGURE 3:**
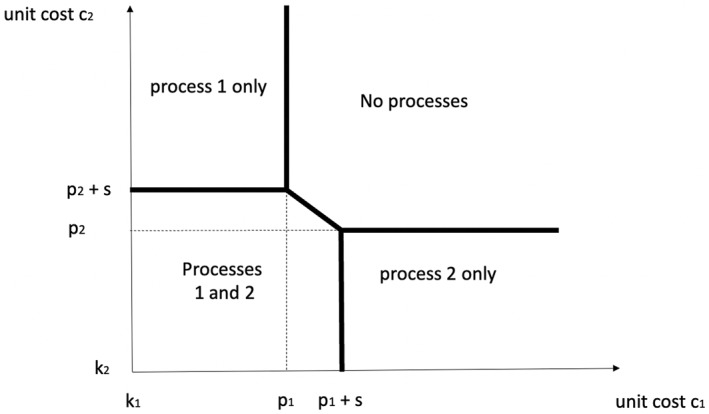
Non‐bundled payment.

Notice that each provider has never an incentive to provide only one of the two processes based on financial ground. This would *not* be the case in a system where the payment is not bundled. To see this, suppose that the purchaser pays a price $p_{1}$ every time process 1 is provided, and $p_{2}$ every time process 2 is provided, where each of these prices are above the minimum provider costs $\left(p_{1}>k_{1},p_{2}>k_{2}\right)$. Under these payment arrangements, three possible scenarios arise. In the first one, the hospital provides only process 1 if providing process 1 is profitable, $\pi_{1}>0$, and providing process 1 is more profitable than providing both processes, $\pi_{1}>\pi_{12}$. These two inequalities reduce to $c_{1}<p_{1}$ and $c_{2}>p_{2}+s$. In the second scenario, the hospital provides only process 2 if it is profitable to do so, $\pi_{2}>0$, and if it is more profitable than providing both processes, $\pi_{1}>\pi_{12}$. These two inequalities reduce to $c_{2}<p_{2}$ and $c_{1}>p_{1}+s$. Finally, the hospital provides both processes if it is more profitable to provide both processes than just one of the two processes, that is $\pi_{12}>\pi_{1}$ and $\pi_{12}>\pi_{2}$, or $c_{2}<p_{2}+s$ and $c_{1}<p_{1}+s$, and the profits of providing both processes are positive, $\pi_{12}>0$. Figure 3 illustrates the solution. As intuitively expected, hospitals with relatively low cost of process 1 and high cost of process 2 provide only process 1. Hospitals with high cost for both processes provide none, and providers with relatively low costs provide both. It is still the case that higher cost synergies increase the number of providers who respond to the financial scheme, for a given pair of prices.

**FIGURE 4:**
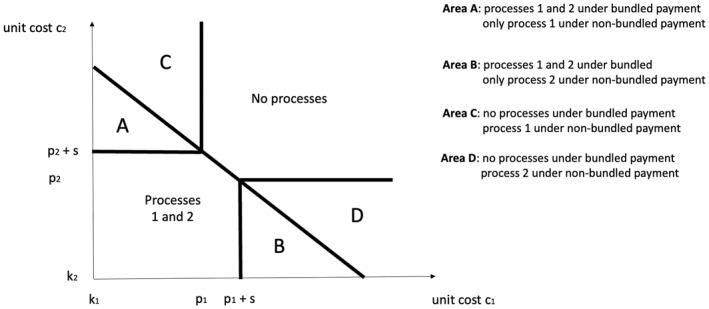
Comparison of bundled and non‐bundled payment.

To further compare the solutions under a bundled payment with a system when payment is not bundled, in Figure 4 we set $P=p_{1}+p_{2}$, so that the bundled price is the sum of the prices when the payment is not bundled and the condition for $\pi_{12}>0$ under bundled payment is $c_{2}<p_{1}+p_{2}-c_{1}+s$. Figure 4 illustrates that providers that fall in areas A and B provide both processes under bundled payment, while only one of the two processes when the payment is not bundled. Therefore, in some cases, the bundled price increases the scope of providing additional care to patients. Conversely, providers in areas C and D provide no processes under bundled payment, but provide one of the two processes when one of the two bundles is not provided. Therefore, in other cases, the bundled price reduces the scope of providing care to patients. The comparison again highlights the strong financial incentive given by the bundled payment to provide “all” or “nothing”.

In summary, our model highlights that there are scenarios where providers are more likely to improve across all processes under a bundled payment. This relates to our empirical analysis, which tests how the introduction of a bundled payment affects performance across different care processes. The model also highlights that if the cost of one of the processes is particularly high for a provider, the bundled payment is not biting and does not give an incentive to improve across any of the processes. Our model is static and assumes that care processes are chosen simultaneously. In practice, some care processes are provided before others. If one of them has been missed by the provider early in the patient pathway (e.g. surgery within 36 h or pre‐operative cognitive assessment for hip fracture patients), this is analytically equivalent to a relative high cost for such process in our static model, and eliminates any financial incentive to provide other care processes later in the patient pathway. Our empirical analysis tests how providers respond in such scenarios.

We conclude the comparison by discussing possible effects on patient health benefits under different payment schemes. To illustrate the key effects at work, consider the special case where the number of providers under area A is the same as under area C, while the number of providers under area B is the same as under area D. In this special case, the total payment of the funder to providers involves the same spending given the assumption that $P=p_{1}+p_{2}$. It is straightforward to show that if patient benefits present no synergies, so that $b_{12}=b_{1}+b_{2}$, then total patient benefit is also the same. It is only if patients benefits present synergies across care processes, so that $b_{12}>b_{1}+b_{2}$, then total patient benefit will be higher under bundled payment. This illustrates one possible benefit of bundled payment, where synergies in benefits (in addition to costs) are advocated as a rationale for providing different care processes under the same payment.^11^

## 5 Data

Our empirical analysis is based on audit data from the National Hip Fracture Database (NHFD) for England and Wales for the period April 2008 to March 2015. The NHFD collects information on care quality for patients aged 60 or over who are admitted to a public NHS hospital with a fragility hip fracture.^12^ We use the recorded information to derive binary indicators of BPT achievement (overall, and by criterion) for each patient in accordance with the BPT payment rules. We define the overall BPT achievement measure as meeting seven criteria in the period from April 2008 to March 2012 and eight criteria in the period from April 2012 to March 2015. The indicator for the pre‐operative cognitive function assessment (BPT4) is included in the overall measure from April 2012 onwards, when it was first used for BPT payment purposes. The indicator for post‐operative cognitive function assessment (BPT5) was not collected in the NHFD prior to April 2011 and is, therefore, excluded from all analyses.

The NHFD provides information on patients' socio‐demographic characteristics including age (recoded to 5‐year age brackets), sex, and admission source. The latter gives an indication on patients' location at the time of the fracture: already in the hospital, in a care home, in a rehabilitation center, or at home. NHFD further provides information on patients' clinical characteristics, including their predicted operative risk, as measured by the American Association of Anesthesiologists (ASA) physical status classification system. Values range from one for a healthy patient to five for a patient who is not expected to survive. Values two to four detail the progressive severity of systemic disease. Further variables describing patients' medical condition include fracture type (based on the anatomical location of the fracture), which is coded as either intracapsular or extracapsular, and patient‐reported level of mobility before the fracture. The latter can take four levels: freely mobile, some mobility, no outdoor mobility and no functional mobility. The socio‐demographic and medical characteristics are used in our empirical analysis to adjust for differences in case‐mix across providers following the risk‐adjustment methodology developed by the Royal College of Surgeons (Tsang and Cromwell 2014).

The initial sample includes 334,850 observations, out of which 317,574 (95%) are in England and 17,276 (5%) in Wales.^13^ In the main analysis we exclude observations with any of the explanatory variables recorded as “unknown” or missing. This applies to 55,697 (17.5%) observations in England, and 3255 (18.8%) in Wales, reducing the sample to 286,265 patients, of which 15,092 were treated in Wales. The original coding for the BPT variables is missing when the relevant BPT is not achieved and thus we code missing as zeros (i.e. not achieved). Appendix Table A1 reports the number of hospitals and patients in our estimation sample by year and country. Participation in the audit is voluntary and has increased steadily since its inception (from 5 hospitals to 13 in Wales; from 105 to 165 in England), reaching full coverage of eligible hospitals in both countries by 2012/13.

Table 1 provides descriptive statistics of patient characteristics for both countries. In England, the average age of patients is 82.6 years, with the vast majority (73.1%) being female. The bulk of patients (77.9%) are admitted from residential homes. More than half of all patients are classified as having severe systematic disease (ASA score 3) with approximately 11.8% facing constant threat to life (ASA score 4 or 5). 35.8% of patients report being freely mobile before the fracture, with 2.2% reporting no functional mobility. Patients characteristics are similar for Wales.

**TABLE 1:** Descriptive statistics of patient characteristics.

Patient characteristic	Mean/%	
	Wales	England
	(*N* = 15,092)	(*N* = 271,173)
Age (in years)	82.2	82.6
Female sex	72.2%	73.1%
Male sex	27.8%	26.9%
Admission source		
Hospital	4.5%	3.5%
Long‐term/nursing care home	15.4%	17.4%
Residential home	78.6%	77.9%
Other	1.6%	1.2%
ASA grade		
1 (Normal healthy patient)	2.7%	2.6%
2 (Mild systemic disease)	30.8%	30.9%
3 (Severe systemic disease)	53.8%	54.7%
4 (Severe systemic disease, constant threat of life)	12.1%	11.4%
5 (Moribund, not expected to survive)	0.6%	0.4%
Pre‐fracture mobility		
Freely mobile before operation	35.2%	35.8%
Full or partial outdoor mobility	25.4%	26.3%
Some indoor mobility	37.3%	35.7%
No functional mobility	2.0%	2.2%
Fracture type		
Intracapsular	57.9%	59.3%
Extracapsular	42.1%	40.7%

*Note:* Data are pooled over the entire study period April 2008 to March 2015.

Table 2 describes hospital performance in each of the nine domains incentivized by the BPT. Two domains with very sharp improvements are BPT2 “Shared care across specialties”, and BPT3 “Multidisciplinary care protocol”. In England, they increase from 5% to 7% in 2009 to 74% and 76%, respectively in 2010, when the incentive scheme was introduced. Similarly, BPT6 “Peri‐operative geriatric assessment” and BPT7 “Geriatrician‐led multidisciplinary rehabilitation” improve from 2% to 8%–58% and 88%, respectively. In contrast, in Wales BPT2 improved only from 3% to 17% between 2009 and 2010, and BPT6 from 3% to 28%, while the improvement was more marked for BPT3 (from 11% to 58%) and BPT7 (from 20% to 76%). For other BPT domains the improvement was more gradual in England.

**TABLE 2:** Proportion of patients receiving BPT care.

Criterion	Financial year						
	2008	2009	2010	2011	2012	2013	2014
England							
Care bundle: All criteria met	0.00	0.01	0.34	0.52	0.61	0.66	0.67
1: Surgery within 36 h	0.54	0.57	0.67	0.72	0.76	0.77	0.77
2: Shared care across specialties	0.01	0.05	0.74	0.88	0.94	0.97	0.98
3: Multidisciplinary care protocol	0.02	0.07	0.76	0.90	0.95	0.98	0.97
4: Pre‐op cognitive function assessment	0.66	0.63	0.62	0.72	0.99	1.00	1.00
6: Peri‐op geriatric assessment	0.00	0.02	0.58	0.75	0.84	0.88	0.90
7: Geriatrician‐led multidisciplinary rehab	0.02	0.08	0.88	0.92	0.94	0.96	0.97
8: Secondary prevention including falls	0.52	0.64	0.81	0.92	0.96	0.97	0.98
9: Bone health assessment	0.68	0.75	0.88	0.94	0.96	0.97	0.98
Wales							
Care bundle: All criteria met	0.00	0.00	0.06	0.03	0.02	0.01	0.01
1: Surgery within 36 h	0.56	0.60	0.59	0.62	0.65	0.65	0.64
2: Shared care across specialties	0.00	0.03	0.17	0.15	0.19	0.15	0.22
3: Multidisciplinary care protocol	0.02	0.11	0.58	0.75	0.77	0.74	0.63
4: Pre‐op cognitive function assessment	0.71	0.72	0.54	0.49	0.70	0.94	0.92
6: Peri‐op geriatric assessment	0.01	0.03	0.28	0.29	0.27	0.32	0.41
7: Geriatrician‐led multidisciplinary rehab	0.03	0.20	0.76	0.88	0.87	0.84	0.89
8: Secondary prevention including falls	0.55	0.43	0.57	0.69	0.71	0.68	0.68
9: Bone health assessment	0.90	0.80	0.90	0.90	0.84	0.84	0.84

*Note:* Financial years run from 1st April to 31st March of the following calendar year.

## 6 Methods

### 6.1 Effect of the Policy on Performance

The aim of our main analysis is to establish a causal link between the introduction of the fragility hip fracture BPT and subsequent changes in the delivery of the incentivized care bundle for hip fracture patients in England. Observed improvements in care quality following the start of the payment policy may be confounded by external effects such as changes in the healthcare production technology, preferences and beliefs among clinical staffs, or demography. We therefore employ a DID approach with patients admitted to English hospitals forming the treatment group and those admitted to Welsh hospitals being in the control group.^14^ Both healthcare systems have similar organizational characteristics, serve similar patient populations, and are subject to the same clinical guidelines (see Section 3). Staffing levels of ortho‐geriatricians, a key input required to meet several BPT criteria, were also comparable prior to the introduction of the payment policy (NHFD 2010). Hence, observed care quality in Wales during the post‐policy period can serve as a credible counterfactual estimate for England.

Our base model takes the following form:

(1) $$Y_{\mathrm{iht}}=\alpha +\theta \left(England_{i}*D_{t}\right)+X_{i}^{\prime }\delta +v_{t}+v_{s}+v_{h}+\epsilon_{\mathrm{iht}}$$

where $Y_{\mathrm{iht}}$ is a dummy variable equal to 1 if the patient i in hospital h in month t fulfills all^15^ BPT criteria and 0 otherwise. $D_{t}$ is a dummy variable equal to 1 in the post‐policy period (from April 2010 to March 2015), and equal to 0 in the pre‐policy period (from April 2008 to March 2010). $England_{i}$ is a dummy variable equal to 1 if patient i is treated in England (the treatment group) and equal to 0 if treated in Wales (the control group). $v_{t}$ is a vector of indicators for each financial year in the study (2009/10 to 2014/15 with reference category 2008/9). $v_{s}$ is a vector of calendar months (January to December, with reference category April) to adjust for seasonality. $v_{h}$ is a vector of hospital fixed effects. $X_{i}$ is a vector of patient characteristics, α is the intercept and $\epsilon_{\mathrm{iht}}$ is the error term. We estimate (1) as a linear probability model with standard errors clustered at the hospital level. The key coefficient of interest is θ, which measures the average treatment effect on the treated (ATT) patient population over the post‐policy period.

The size of the bonus payment increased in year 2 (2011/12), and then again in year 3 (2012/13). We implement a version of our base model that captures differential responses over time, that is

(2) $$Y_{\mathrm{iht}}=\alpha +\sum_{k=2010/11}^{\mathrm{2012∕13}}\theta_{k}\left(England_{i}*Period_{k}\right)+X_{i}^{\prime }\delta +v_{t}+v_{s}+v_{h}+\epsilon_{\mathrm{iht}}$$

where $Period_{k}$ are binary indicators for the three post‐policy periods with differing incentive payments and the $\theta_{k}$ coefficients measure the corresponding ATTs.

The level of pre‐policy achievement differs considerably across process measures which implies differential scope for improvement. English hospitals could have an incentive to focus on improving those process measures where pre‐policy achievements were lowest in order to deliver the full care bundle. Furthermore, process measures are likely to differ in marginal cost and patient benefit, both of which are unobservable to us but may affect how providers prioritize these process measures. To explore heterogeneous effect of the policy across different process measures, we re‐estimate the models in Equations (1) and (2) for each of the BPT criteria separately. Building on this, we also test whether the policy had an impact on the number of criteria met, with the understanding that providers in Wales might still aim to improve on the care they provide, but with less emphasis on achieving all the criteria.

### 6.2 Robustness Checks and Sensitivity Analyses

**FIGURE A1:**
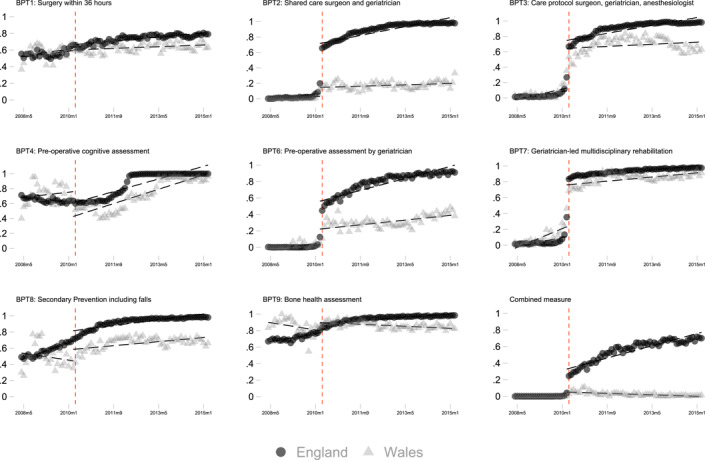
BPT attainment over time for England and Wales.

The validity of the DID approach relies on the parallel trends assumption, that is the outcomes of interest would develop similarly in both groups in the absence of the policy intervention. We explore this assumption in two ways. First, we carry out a a visual inspection of the pre‐policy trends (see Appendix Figure A1). Second, we test the assumption empirically by using pre‐policy data (2008/9–2009/10) to estimate the following model that allows for country‐specific trends:

(3) $$Y_{\mathrm{iht}}=\alpha +\sum_{k=1}^{7}\beta_{k}Quarter_{k}+\sum_{k=1}^{7}\tau_{k}\left(England_{i}*Quarter_{k}\right)+X_{i}^{\prime }\delta +v_{s}+v_{h}+\epsilon_{\mathrm{iht}}$$

where $Quarter_{k}$ are binary variables taking value of 1 if the patient was treated in quarter k(k=1,…,7), and 0 otherwise. We use quarterly dummies rather than year dummies (as used in the main regression) to add granularity and so better control for short term changes in the pre‐policy period. The reference group is the first quarter. The coefficients $\tau_{k}$ capture the difference in the pre‐policy trends between the two countries. The null hypothesis for the parallel trends assumption is $H_{0}:\tau_{k}=0$. Failure to reject this assumption provides reassurance that the parallel trends assumption holds.

We perform three further sensitivity analyses of our base specifications Equations (1) and (2). First, we control for the increasing number of hospitals reporting to the NHFD by estimating our specifications on data from a balanced panel of hospitals that have participated in the audit in all financial years since 2008/9 and have treated at least 30 patients per year. Second, we estimate both specifications with a limited set of control variables (age, sex, and fracture type) which are recorded for all patients in the NHFD and drop other control variables where information is missing for some patients. This increases the estimation sample to 334,835 patients at the possible expense of a less comprehensive risk‐adjustment. Finally, we use the seven initial BPT criteria that have been in place since April 2010 (i.e. excluding BPT4 and BPT5) to define achievement in all years of the sample.

### 6.3 Sequential Decision‐Making

Payment of the BPT bonus is conditional on providing all of the incentivized measures. Therefore, the financial incentive to providers to deliver any additional processes drops to zero if at least one other process measure has been missed already. We exploit the sequential nature of care processes within the hip fracture care pathway to study how missing one or more BPT criteria, and therefore forgoing the bonus payment, affects provider behavior with respect to any remaining criteria that are yet to be met. Assuming that achieving BPT criteria is costly to the provider one would expect them to exert less effort to meet criteria once at least one criterion has been missed. The exact order of events is unknown to us. However, we can categorize BPT criteria into two groups: those where processes are expected to take place before surgery (BPT1, BPT3, BPT4, BPT6) and those were processes take place after surgery (BPT7, BPT8, BPT9). The allocation of BPT criteria to these two groups was informed by clinical expert opinion. We use this classification to estimate the effect of failing to meet at least one preoperative criterion on the propensity of meeting *all* postoperative criteria using the following model:

(4) $$Post_{\mathrm{ith}}=\alpha +\gamma Pre_{\mathrm{ith}}+\theta \left(England_{i}*Pre_{\mathrm{ith}}\right)+{X_{i}}^{\prime }\delta +v_{t}+v_{s}+v_{h}+\epsilon_{\mathrm{iht}}$$

where $Post_{\mathrm{ith}}$ is a binary indicator taking the value of 1 if all BPT criteria related to post‐surgical care processes were attained for patient i in hospital h at time t, and 0 otherwise. $Pre_{\mathrm{ith}}$ is a binary indicator equal to 1 if one or more of the pre/mid‐surgery BPT criteria are missed and 0 otherwise. The coefficient θ captures the difference in response between England and Wales if a pre‐surgery criteria is missed. A positive estimate of θ suggests that English hospitals are more likely than Welsh hospitals to achieve post‐surgery criteria once an earlier criterion is missed, which, in turn, implies that the existence of the BPT incentive policy has positive spillovers on process measures even when these are no longer incentivized. Conversely, a negative estimate of θ suggests that English providers respond strategically to the loss of incentive and reduce their effort toward the level exerted by Welsh hospitals. We run Equation (4) as a linear model with standard errors clustered at the hospital level. The pre‐surgery criteria rely heavily on the involvement of geriatricians and have attainment level close to zero in both countries prior to the introduction of the BPT (see Appendix Table A2). We therefore limit this analysis to the period after the BPT introduction (i.e. April 2010 onwards). Furthermore, note that BPT2 (shared care between surgeon and geriatrician) is excluded from analysis because achievement levels are consistently low in Wales during the study period. This results in very low proportions of patients for whom all pre‐surgery criteria are met. $\left(Pre_{\mathrm{ith}}=1\right)$.^16^ This analysis relies on the assumption that providers do not decide to meet the pre‐surgery criteria based on their expectations about the likelihood of meeting the post‐surgery criteria. Considering the nature of the criteria and the uncertainty about meeting the subsequent criteria (as they are days/weeks away), this seems to be a reasonable assumption.

## 7 Results

### 7.1 Effect of the BPT on Delivery of Care Bundles

**FIGURE 5:**
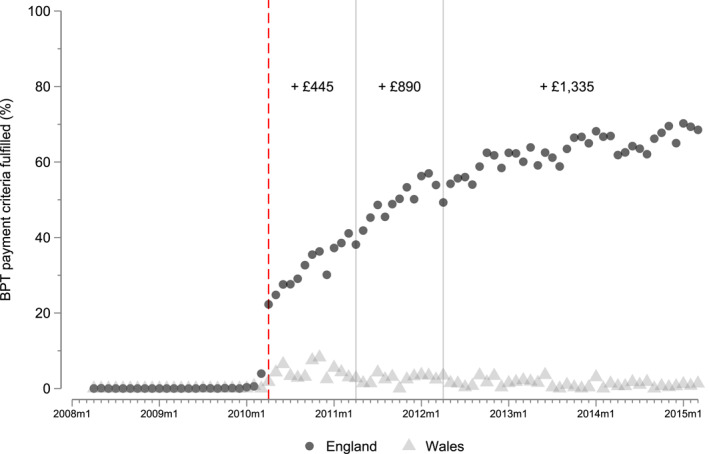
BPT achievement in England and Wales over time.

Figure 5 shows the proportion of patients receiving the full care bundle in England and Wales over time, where achievement is defined as having met 7 criteria in the period up to March 2011 and as having met 8 criteria in the subsequent period. Vertical lines indicate changes in the size of the bonus paid to providers in England that deliver the full care bundle. Achievement is very low in both countries during the pre‐policy period. After the introduction of the BPT payment policy in April 2010, care processes improve rapidly in England but remain low in Wales.

Table 3 presents results of the main DID analyses both in terms of the average effect of the policy (Equation 1) and for each of the 3 years in the post‐policy period (Equation 2). Our key results indicate a large and statistically significant increase in the probability of receiving the incentivized care bundle of 52.5% points (pp) (*p* <0.01) over the post‐policy period; 26.6 pp in the first period (April 2010 to March 2011), 46.7 pp in the second period (April 2011 to March 12) and 60.6 pp in the third period (April 2012 to March 15) (all *p* <0.01).

**TABLE 3:** Regression estimate of the effect of the BPT payment policy on the probability of delivering the incentivized care bundle.

	(1)		(2)	
	Single post‐policy period		Multiple post‐policy periods	
	Estimate	SE	Estimate	SE
Policy effect				
Average	0.525***	0.054		
April 2010–March 2011			0.266***	0.078
April 2011–March 2012			0.467***	0.056
April 2012–March 2015			0.606***	0.042
Age groups				
60–64 (reference)				
65–59	0.013**	0.006	0.013**	0.006
70–74	0.030***	0.006	0.030***	0.006
75–79	0.040***	0.005	0.040***	0.005
80–84	0.049***	0.005	0.049***	0.005
85–89	0.053***	0.006	0.053***	0.005
90+	0.062***	0.006	0.062***	0.006
Gender				
Female (reference)				
Male	−0.020***	0.002	−0.020***	0.002
Admission source				
Hospital (reference)				
Care home	0.091***	0.008	0.090***	0.008
Residential home	0.065***	0.008	0.064***	0.008
Other	0.074***	0.011	0.073***	0.011
ASA Grade				
ASA Grade 1 (reference)				
ASA Grade 2	−0.016**	0.006	−0.016**	0.006
ASA Grade 3	−0.051***	0.007	−0.052***	0.006
ASA Grade 4	−0.147***	0.008	−0.148***	0.008
ASA Grade 5	−0.319***	0.024	−0.319***	0.024
Mobility				
Full mobility (reference)				
Some outdoor mobility	−0.001	0.003	−0.001	0.003
Some indoor mobility	0.009**	0.003	0.008**	0.003
No functional mobility	−0.025***	0.007	−0.025***	0.007
Fracture type				
Extracapsular (reference)				
Intracapsular	0.021***	0.002	0.021***	0.002
Hospital fixed effects	X		X	
Time (month) fixed effects	X		X	
Patient characteristics	X		X	
Number of hospitals	186		186	
Number of patients	286,265		286,265	

*Note:* Model 1 estimates the average effect of the BPT policy over the first 5 years after the policy introduction (based on Equation 1). Model 2 estimates separate effects for the three post‐policy periods that coincide with changes in the size of the bonus payment (based on Equation 2). Note that the third period covers three financial years, whereas the other periods cover one financial year each. Standard errors (SEs) are clustered at the hospital level.

*** *p* < 0.01.

** *p* < 0.05.

*p < 0.1.

We do not find evidence of a violation of the parallel trends assumption that underpin these DID analyses. All of the estimates for the difference in trends in the pre‐policy period, presented in Table 4 are small (between −0.001 and 0.019 pp per quarter). With the exception of the last quarter, all of the estimates are also statistically insignificant. This finding is confirmed by visual inspection of the data (Figure 5). The coefficient in the last calendar quarter (January 2010–March 2010) prior the start of the policy is statistically significant, indicating a potential anticipation effect. However, the effect is still small in comparison to the overall effect of the policy, and unlikely to bias results.

**TABLE 4:** Differences in achievement of care bundle between England versus. Wales by quarter of pre‐policy period.

Quarter	Estimate	SE
Apr08–June08	*Reference*	
July08–Sept08	−0.001	0.001
Oct08–Dec08	−0.001	0.001
Jan09–Mar09	−0.001	0.001
Apr09–June09	−0.001	0.001
July09–Sept09	−0.000	0.001
Oct09–Dec09	0.001	0.001
Jan10–Mar10	0.019***	0.004
Joint test	0.017**	0.006
Joint test minus anticipation period	−0.023	0.004
Hospital fixed effects	X	
Patient characteristics	X	
Number of hospitals	163	
Number of patients	37,822	

*Note:* We test the parallel trends assumption using data from the pre‐policy period (April 2008 to March 2010) and quarterly dummies. Standard errors (SEs) are clustered at hospital level.

*** *p* < 0.01.

** *p* < 0.05.

*p < 0.1.

Our sensitivity analyses, presented in Table 5, confirm the robustness of our main results. The estimate of the policy effect reduces to 50.7 pp when we base our analysis on a balanced panel of hospitals that have participated in the clinical audit in all years since the start of our dataset. Although this suggests that early contributors to the NHFD performed worse than average, any selection bias is likely to be of little practical importance. Furthermore, the overall achievement rate of the early contributors was close to zero in the pre‐policy period, which is likely to be the case for hospitals joining the NHFD at a later point as well. We also find that controlling for a limited set of patient characteristics with low levels of missing data (only age, sex, and fracture type) has limited effect on the estimated policy effect (53.1pp), which suggests that hospitals may face a relatively similar case‐mix of patients.^17^ Exclusion of one criterion (BPT4: “pre‐operative mental health assessment”) from the overall measure increases marginally the estimated effect of the policy (55.4 pp), mainly as the achievement rates for this criterion were already high in both countries prior to its inclusion in the BPT incentive. Finally, model (4) excludes the 6 months prior to the introduction of the BPT policy to account for possible anticipation. This reduces the impact of the policy by 1.5pp (52.5pp vs. 51.0pp) which suggests that hospitals may only have made minor adjustments to their operating procedures once they became aware of the upcoming policy change.

**TABLE 5:** Results of sensitivity analyses.

Policy effect	(1)		(2)		(3)		(4)	
	Balanced panel		Limited case‐mix adjustment		Subset of 7 BPT criteria		Excluding anticipation period (6 months)	
	Estimate	SE	Estimate	SE	Estimate	SE	Estimate	SE
Average	0.508***	0.068	0.531***	0.036	0.554***	0.075	0.510***	0.066
April 2010–March 2011	0.271**	0.111	0.279***	0.060	0.369**	0.127	0.253**	0.088
April 2011–March 2012	0.450***	0.076	0.471***	0.043	0.570***	0.101	0.454***	0.066
April 2012–March 2015	0.589***	0.047	0.615***	0.028	0.594***	0.055	0.593***	0.052
Hospital fixed effects	X		X		X		X	
Time (month) fixed effects	X		X		X		X	
Patient characteristics	X		X*		X		X	
Number of hospitals	87		186		186		186	
Number of observations	161,064		347,447		286,265		274,916	

*Note:* Model 1 uses a balanced panel of hospitals who reported to the NHFD in all years during our study period. Model 2 adjusts for a limited set of case‐mix variables (age, sex, fracture type) for which information was available for the full sample. Model 3 excludes BPT4 (“Pre‐operative cognitive function assessment”) from the definition of the care bundle. Model 4 excludes observations in the 6 months prior to policy start, when providers may have been aware of impeding changes to payment modalities (“anticipation period”). Standard errors are clustered on hospital level.

*** *p* < 0.01.

** *p* < 0.05.

* *p* < 0.1.

### 7.2 Effect of the BPT on Individual Care Processes

The analyses at the level of care bundles hide some important heterogeneity across the criteria that constitute the care bundle (Table 6). The BPT has the largest impact on process measures related to geriatrician involvement (BPT2, BPT3, BPT6, BPT7), ranging from 56.2 pp for shared care across specialities to 17.2 pp for geriatrician‐led multidisciplinary rehabilitation. These results resonate with Neuburger et al. (2017), who reported a large increase in the number of full‐time equivalent geriatricians working in NHS hospitals in England following the start of the BPT payment policy.^18^ The policy also increased the proportion of patients with bone health assessment carried out by 23.3 pp, while the policy impact is generally less pronounced for the remaining BPT criteria. This is due to either i) similar improvements being observed in both countries, or ii) initial rates being already high prior to the policy (see Table 2). The BPT policy increased the probability of receiving surgery within 36h (BPT1) by 13.1 pp. The effects on secondary prevention (BPT8) and pre‐operative cognitive assessment (BPT4) are both small (0.3 pp and 4.5pp, respectively) and not statistically significant. Taken together, the policy increased the number of criteria met by 2.0.

**TABLE 6:** Estimated policy effects by BPT criterion.

	(1)		(2)					
	Full post‐policy period		April 2010 to March 2011		April 2011 to March 2012		April 2012 to March 2015	
	Estimate	SE	Estimate	SE	Estimate	SE	Estimate	SE
Criterion met								
BPT1: Surgery within 36 h	0.131***	0.028	0.096***	0.025	0.121***	0.024	0.143***	0.035
BPT2: Shared care across specialties	0.562***	0.169	0.429**	0.176	0.540***	0.170	0.602***	0.164
BPT3: Multidisciplinary care protocol	0.240*	0.125	0.202	0.143	0.209	0.132	0.259**	0.129
BPT4: Pre‐op cognitive function test	0.003	0.094	n/a		n/a		0.003	0.094
BPT6: Peri‐op geriatric asses.	0.428***	0.066	0.295***	0.108	0.407***	0.090	0.467***	0.055
BPT7: Multidisciplinary rehab	0.172**	0.071	0.216**	0.110	0.139**	0.067	0.170**	0.074
BPT8: Falls prevention	0.045	0.125	0.072	0.074	−0.002	0.131	0.052	0.141
BPT9: Bone health assessment	0.233***	0.061	0.126***	0.040	0.210***	0.056	0.266***	0.074
Total number of criteria met								
Count of criteria	2.057***	0.278	1.517***	0.449	1.811***	0.291	2.262***	0.272

*Note:* Model 1 estimates the average effect of the BPT policy over the first 5 years after the policy introduction (based on Equation 1). Model 2 estimates separate effects for the three post‐policy periods that coincide with changes in the size of the bonus payment (based on Equation 2). Note that the third period covers three financial years, whereas the other periods cover one financial year each. Standard errors (SEs) are clustered at the hospital level.

*** *p* < 0.01.

** *p* < 0.05.

* *p* < 0.1.

We can also compare how the correlation between different criteria that involve geriatricians has evolved over time before and after policy in the treatment and control group. In England (see Table A5 in the Appendix), in the pre‐policy period the four criteria involving geriatricians (BPT 2,3,6,7) had a correlation that varied from 0.31 to 0.80. Instead, the correlation was much lower between criteria that involved geriatriacians and those not involving geriatricians. For example, the correlation between BPT2 and BPT9 (bone health assessment) was only 0.04. This supports the hypothesis that there are synergies between different tasks provided by geriatricians. In the post‐policy period, these correlations generally increased. For example, the correlation between BPT2 (shared care by surgeon and geriatrician) and BPT6 (perioperative assessment by geriatrician) increased from 0.42 to 0.77, and between BPT2 and BPT7 (geriatrician‐led multidisciplinary rehabilitation) increased from 0.31 to 0.67. But the correlation between BPT2 and BPT9, which was low pre‐policy, also increased to 0.64 due to the overall increase in performance across criteria. For Wales (Table A6), the correlations between criteria involving geriatricians are low and even negative. For example, the correlation between BPT2 and BPT6 is −0.03 and between BPT2 and BPT7 is −0.25 in the pre‐policy period. There is also low correlation between criteria involving geriatricians and those not involving geriatricians. For example, the correlation between BPT2 and BPT9 is −0.25. In the post‐policy period these correlations remain low or negative.

As can bee seen in Table 6, for most criteria the magnitude of the effect is increasing over time, with the largest effects observed during the period from 2012/13 to 2014/15 for 5 out of 8 criteria. However, as in the case of the overall measure, the rate of change is slowing over time, with the largest absolute increase observed in the first year of the policy for all the criteria. This suggests that hospitals responded strongly at the start of the policy, despite the lower BPT bonus in the first year relative to the following years.

**FIGURE 6:**
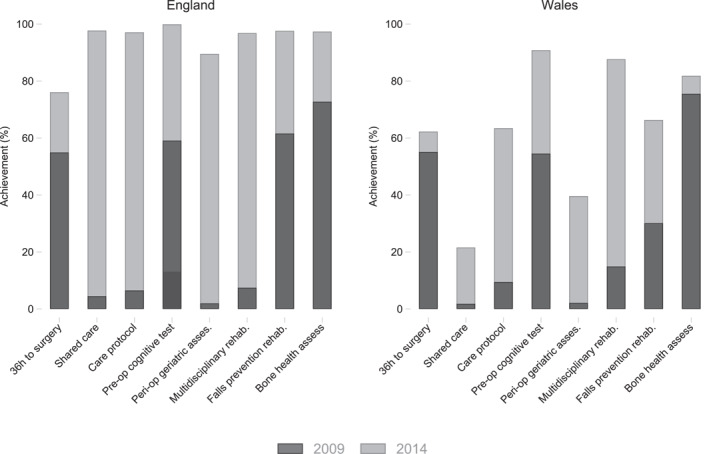
Achievement of BPT criteria in England and Wales, 2009/10 and 2014/15.

Figure 6 shows that in the England all BPT measures reached similar levels by 2014/15, regardless of the initial values before the start of the policy in 2009/10.^19^ Conversely, despite similar starting levels as in England, Wales shows much larger dispersion between the BPT achievement levels in 2014/15. The wide‐ranging improvement in England is likely due to the bundled element of the BPT, as it requires the hospitals to improve on all segments to receive the additional payment, as also highlighted by our theoretical model in Section 4.

Appendix Table A3 reports the results of the parallel trends test for individual criteria and the number of criteria met (see also Appendix Figure A1 for a visual representation). There is no strong evidence of systematic deviations in trends between England and Wales prior to the start of the BPT payment policy.

### 7.3 Sequential Decision‐Making

We next look at how providers respond to the loss of financial incentive once they have missed at least one criterion for a given patient (Table 7). As previously shown, English hospitals increased their achievement across nearly all BPT criteria following the start of the BPT payment policy in April 2010 and, consequently, are more likely to meet post‐operative criteria than hospitals in Wales in the post‐policy period. Providers in both countries are significantly less likely to achieve post‐operative criteria once at least one pre‐operative criterion has been missed. Interestingly, we do not find a statistically significant interaction between being treated in England and the effect of having missed one or more pre‐operative criteria, suggesting that English hospitals may not be changing their efforts to the level of Welsh hospitals once the incentive payment is no longer attainable. This may arise, for example, because of imperfect information flows between health personnel. However, we cannot observe such behavior in our data and can therefore not test this hypothesis. Analysis by year generates similar results (see Table A4 in the Appendix), although we do identify a positive and significant coefficient on the interaction term for the last financial year of our time series (2014/15).

**TABLE 7:** The effect of failing one or more preoperative criteria on the probability of meeting the set of post‐operative criteria.

	(1)		(2)		(3)	
	Observed		Without FE		With FE	
	Estimate	SE	Estimate	SE	Estimate	SE
Pre‐criteria failed	−0.289***	0.086	−0.265***	0.095	−0.137***	0.027
England	0.156**	0.074	0.155**	0.097	n/a	
Pre‐criteria failed × England	0.060	0.088	0.094	0.096	0.015	0.029
Hospital fixed effects					X	
Time (month) fixed effects			X		X	
Patient characteristics			X		*X*	
Number of hospitals	183		183		183	
Number of observations	298,305		248,443		248,443	

*Note:* Estimated during post‐policy period April 2010–March 2015. Analysis excludes BPT2 from pre‐surgery measures due to low achievement in Wales. Standard errors are clustered at hospital level.

*** *p* < 0.01.

** *p* < 0.05.

*p < 0.1.

## 8 Discussion

Care bundling is intended to affect both the overall level (via the P4P component) and the distribution of efforts (via the “*all‐or‐nothing*” bundling component) that providers exert. Our results show that the BPT policy increased the delivery of the full care bundle for hip fracture patients by 52.5pp in the first 5 years following implementation. Similar results are obtained when varying the way in which we adjust for case‐mix, select the sample of hospitals or criteria to be analyzed, or account for potential anticipation effects. We also identified considerable heterogeneity in the response across different criteria that make up the care bundle. The policy effect was largest for the four criteria associated with geriatrician involvement in the care, with smaller improvement observed in other areas. The sizable difference between the overall policy effect and the effect of individual criteria suggests the English providers responded strategically to the scheme, by improving across all dimensions to the level required for the bonus payment.^20^ While the absolute achievement rates varied across criteria in both England and Wales prior to the introduction of the BPT, by 2014/15 English providers achieved comparable achievement rates across all of the criteria, regardless of the initial performance. In contrast, Welsh providers improved individual care processes in a less systematic way. The difference in response across countries indicates that the bundled element of the scheme focused the attention of English providers on all of the care processes, rather than individual tasks.

The “*all‐or‐nothing*” nature of the care bundling incentive creates a dynamic decision problem for providers. Once at least one criterion has been missed, providers have no direct financial incentive anymore to proceed with the other criteria (although they may have altruistic or professional motivations). If providers are responsive to the change in incentive, purchasers may need to factor in the temporal order of incentivized activities and their relative importance for achieving the overarching goal (e.g. increase patient health benefit) when deciding whether or not to bundle incentives. If, for example, early activities are difficult to achieve or driven by chance *and* they only make a relatively small contribution toward the overarching goal, it may be better to exclude them from the incentivized care bundle and reward providers separately for these activities instead. However, our empirical analysis fails to provide evidence that English hospitals respond to the reduction in incentive once the reward becomes unattainable for individual patients. This suggests that purchasers can rely on some provider inertia when selecting which care processes to bundle into one incentive payment.

Our results suggest the BPT scheme for hip fracture yields better results compared to several other P4P programs (Milstein and Schreyoegg 2016; Mokhtary et al. 2024; Mathes et al. 2019). While it is difficult to pinpoint the reasons for this relative success of the scheme, it may be attributed to specific design features. The bonus size in P4P schemes within the hospital setting is often relatively small (less than 2%–3% of total reimbursement) (Cashin et al. 2014), whereas this scheme operates with a substantially larger bonus, reaching 21% of the total reimbursement of care. Furthermore, the selection of the incentivized criteria relied heavily on clinical input, mirroring the clinical guidance for the optimal treatment of hip fracture patients. Hence the scheme may have given stronger motivation and resources to the physicians to treat the patients according to what they themselves perceived as best practice. This is likely to have helped align incentives between hospital managers and clinicians; thus avoiding a possible conflict between financial and quality incentives identified in the economic literature that may limit behavioral responses (Harris 1977; Galizzi and Miraldo 2011).

## 9 Conclusions

Our study shows that P4P can be effective in increasing the provision and, more broadly, the quality of health care. Previous studies have shown that P4P has had limited impact within the context of secondary care, and has only occasionally been more successful within the context of ambulatory or primary care. We have shown that P4P can be successful also in the context of secondary care and highlight different distinct economic features of a specific scheme. First, the scheme incentivized process measures of care as opposed to health outcomes and these process measures were chosen to reflect best practice standards based on clinical evidence and professional consensus. Second, there was just one single payment which was conditioned on a bundle of processes. The simplicity of the scheme combined with the strong financial incentive to provide the care bundle could be a contributing factor to greater provider attention and focus to increase efforts. Third, the size of the bonus was significant, potentially suggesting that the small effects from previous schemes may be driven by the small bonus. Overall, the study supports policies that gradually move away from activity‐based financing in favor of payment models that reward quality directly.

## Conflicts of Interest

The authors declare no conflicts of interest.

## Data Availability

The data that support the findings of this study are available from Royal College of Physicians. Restrictions apply to the availability of these data, which were used under license for this study. Data are available from https://www.nhfd.co.uk/ with the permission of Royal College of Physicians.

## Endnotes

^1^
Examples within secondary care include the Hospital Readmission Reduction Program in the US, and Advancing Quality in the UK.

^2^
Our study does not look at the impact of the BPT on health outcomes as such data are not captured with sufficient coverage in our clinical registry dataset.

^3^
This approach differs from care bundling, as it is not based on several quality measures. Instead, it requires a single criterion/quality measure to be met for a defined proportion of all patients for the hospital to receive the payment.

^4^
In 2014/15, the English NHS spent £2055 per capita on healthcare, whereas the Welsh NHS spent £2083 per capita (Harker 2014).

^5^
The current clinical guideline CG124 details the appropriate management of hip fracture and has been in place since 2011. The fragility hip fracture BPT incentivizes many elements of this guideline.

^6^
Financial years run from first of April to 31st of March of the following year.

^7^
The average HRG base price for hip fracture patients in 2014/15 was £6369 (NHS England 2014). With the average hospital treating 450 eligible patients every year, this represents an average potential bonus of £0.6 M or approximately 0.15% of the average total hospital budget.

^8^
In 2017, the list of BPT criteria was revised and some new criteria were introduced whereas some old ones were removed.

^9^
The NHFD is now commissioned by the Healthcare Quality Improvement Partnership and managed by the Royal College of Physicians as part of the Falls and Fragility Fracture Audit Programme.

^10^
The key insights of the model would be qualitatively similar if we assume that providers are partially altruistic as in Ellis and McGuire (1986). Define α as the degree of altruism, and u as provider payoff function. Then the utility function under bundling would be $u_{12}=P+\alpha b_{12}-c_{12}$, and $u_{1}=\alpha b_{1}-c_{1}$, $u_{2}=\alpha b_{2}-c_{2}$ if just one of the two processes are provided and $u_{0}=0$ if none are provided, where $b_{12}>b_{1}+b_{2}$ if we allow for synergies in benefit. Then, we can identify a similar figure to the one provided in Figure 1 by redefining the *x*‐ and *y*‐axis in terms of $c_{1}-\alpha b_{1}$ and $c_{2}-\alpha b_{2}$. In terms of incentives, the only difference would be that the hospital would have an incentive to provide only process 1 if the cost of process 1 is very small and the cost of process 2 is very large; conversely, it would provide only process 2 only if the cost of process 2 is very small and the cost of process 1 is very high.

^11^
The discussion relies on the assumption that the total number of providers is constant under the two schemes. This is a strong assumption, as the number of providers are likely to differ. However, the additional effects when the number of providers in areas A and C or B and D differ are predictable and depend on the price levels under each scheme, and the shape of the joint density function. A full welfare analysis is beyond the scope of this section, which mostly aims at illustrating provider incentives under different schemes.

^12^
Private provision of hip fracture treatment is not recorded but is likely to be close to zero given the emergency nature of the condition. Private hospitals in the UK focus on selected elective procedures and do not normally have the required capabilities to treat emergency patients.

^13^
Hip fracture incidence rates are comparable across England and Wales (Curtis et al. 2016). Differences in the number of cases reflect difference in the size of the population of both countries.

^14^
The approach of comparing similar geographic regions to test for policy effects has been widely used in literature, for example, comparing England and Scotland (Propper et al. 2008; Farrar et al. 2009) or different US states (Kolstad and Kowalski 2016).

^15^
Seven criteria in financial year 2010/11, and eight criteria from 2011/12 onwards. See Section 5.

^16^
The achievement of all pre‐surgery BPT criteria in Wales between April 2010 and March 2015 is 2.9% when including BPT2 and 15.1% when excluding this criterion (see Table A2).

^17^
Patients sustaining a fragility hip fracture are unlikely to bypass their local hospital (Gutacker et al. 2016), which implies that concerns over endogenous selection are unlikely to apply.

^18^
Qualitative evidence supports the assumption that the BPT led to an increase in geriatricians involved in hip fracture care. McDonald et al. (2012) conducted a mixed‐methods study to evaluate the early stage of the roll‐out of the BPT in England. They note that in their interview “various changes were described which included […] investing additional resources to secure additional staff input from clinicians, particularly in relation to orthogeriatrician time and weekend cover […]” (McDonald et al. 2012, 13).

^19^
While for majority of criteria providers reached very high achievement rates, BPT1 (36h to surgery), seems to be lagging behind. However, this is most likely due to clinical guidelines that state that it can be beneficial to postpone the surgery for some groups of patients (predominantly younger ones) because further stabilization may results in better overall clinical outcome.

^20^
Norton et al. (2018) also found that providers responded strategically to the Hospital Value‐based Purchasing Program in US Medicare by adjusting their efforts according to patients' marginal incentive payments.

## Appendix

**TABLE A1:** Number of hospitals and patients included in the estimation sample, 2008/9 to 2014/15.

Financial year	Hospitals		Patients	
	Wales	England	Wales	England
2008/09	5	105	398	13,930
2009/10	9	152	920	22,574
2010/11	11	166	1582	34,750
2011/12	12	167	2346	43,744
2012/13	13	165	3155	48,122
2013/14	13	165	3303	52,377
2014/15	13	163	3388	55,676

*Note:* The table shows the number of hospitals reporting to the NHFD and the number of patients recorded within NHFD by year and country. The line represents the start of the BPT policy in April 2010.

**TABLE A2:** Number of patients (proportion of total) for which the all pre‐/during and post‐surgical BPT criteria were achieved.

Period	Pre‐surgery		Pre‐surgery excl. BPT2		Post‐surgery	
	England	Wales	England	Wales	England	Wales
Pre‐policy (April 08 to March 10)	169	3	279	3	2000	96
	(0.3%)	(0.1%)	(0.5%)	(0.1%)	(3.8%)	(3.8%)
Post‐policy (April 10 to March 15)	145,326	482	162,024	2484	246,057	7091
	(51.6%)	(2.9%)	(58.0%)	(15.1%)	(87.3%)	(56.9%)

*Note:* “Pre‐surgery” refers to care process that are expected to take place prior to or during the surgery (BPT1, BPT3, BPT4, BPT6). ’Post‐surgery’ refers to care processes that are expected to take place after the surgery (BPT7, BPT8, BPT9).

**TABLE A3:** Empirical test of the parallel trends assumption: individual criteria.

	BPT1		BPT2		BPT3		BPT4		BPT6		BPT7		BPT8		BPT9		Number of criteria	
	Estimate	SE	Estimate	SE	Estimate	SE	Estimate	SE	Estimate	SE	Estimate	SE	Estimate	SE	Estimate	SE	Estimate	SE
Quarter																		
July08–Sept08	−0.091**	0.037	0.007	0.006	0.001	0.005	−0.004	0.073	−0.001	0.002	0.001	0.005	−0.050	0.087	−0.008	0.049	−0.145	0.129
Oct08–Dec08	−0.156***	0.031	0.010*	0.006	−0.006	0.007	−0.129	0.097	−0.001	0.002	−0.012	0.009	−0.157	0.117	−0.064	0.059	−0.515***	0.162
Jan09–Mar09	−0.147	0.095	0.006*	0.004	−0.010	0.013	−0.170	0.183	−0.017	0.015	−0.012	0.018	−0.026	0.123	−0.033	0.084	−0.409*	0.229
Apr09–June09	−0.157	0.115	0.012	0.007	−0.000	0.013	−0.108	0.179	−0.017	0.016	−0.049*	0.030	0.070	0.096	0.071	0.067	−0.179	0.345
July09–Sept09	−0.193*	0.112	0.017*	0.009	−0.001	0.017	−0.197	0.174	−0.015	0.015	−0.059	0.036	0.116	0.095	0.097	0.072	−0.236	0.334
Oct09–Dec09	−0.188	0.136	0.021***	0.008	−0.006	0.029	−0.352*	0.197	−0.005	0.011	−0.049	0.030	0.056	0.133	0.227***	0.086	−0.295	0.418
Jan10–Mar10	−0.195	0.120	0.047	0.056	−0.083	0.145	−0.272	0.208	0.016	0.033	−0.139	0.109	0.062	0.196	0.065	0.072	−0.497	0.521
April10–June10	n/a	n/a	n/a	n/a	n/a	n/a	−0.181	0.219	n/a	n/a	n/a	n/a	n/a	n/a	n/a	n/a	n/a	n/a
July10–Sept11	n/a	n/a	n/a	n/a	n/a	n/a	−0.203	0.229	n/a	n/a	n/a	n/a	n/a	n/a	n/a	n/a	n/a	n/a
Oct10–Dec10	n/a	n/a	n/a	n/a	n/a	n/a	−0.273	0.208	n/a	n/a	n/a	n/a	n/a	n/a	n/a	n/a	n/a	n/a
Jan11–March11	n/a	n/a	n/a	n/a	n/a	n/a	−0.191	0.234	n/a	n/a	n/a	n/a	n/a	n/a	n/a	n/a	n/a	n/a
April11–June11	n/a	n/a	n/a	n/a	n/a	n/a	−0.110	0.236	n/a	n/a	n/a	n/a	n/a	n/a	n/a	n/a	n/a	n/a
July11–Sept11	n/a	n/a	n/a	n/a	n/a	n/a	−0.123	0.248	n/a	n/a	n/a	n/a	n/a	n/a	n/a	n/a	n/a	n/a
Oct11–Dec11	n/a	n/a	n/a	n/a	n/a	n/a	−0.095	0.244	n/a	n/a	n/a	n/a	n/a	n/a	n/a	n/a	n/a	n/a
Jan12–March12	n/a	n/a	n/a	n/a	n/a	n/a	−0.089	0.232	n/a	n/a	n/a	n/a	n/a	n/a	n/a	n/a	n/a	n/a

*Note:* We perform the analysis of to test the pre‐parallel trends for each individual BPT criteria and for the number of criteria met, using the data from the period April 2008–March 2010 and quarterly dummies. We perform the analysis of to test the pre‐parallel trends for BPT4, using the data from the period April 2008–March 2012 and quarterly dummies. The reference category for quarters is April 2008–June 2008. Standard errors are clustered on hospital level.

*** *p* < 0.01.

** *p* < 0.05.

* *p* < 0.1.

**TABLE A4:** The effect of failing one or more pre‐operative criteria on the probability of meeting the set of post‐surgical criteria—by financial year.

	(1)		(2)		(3)		(4)			(5)	
	2010/11		2011/12		2012/13		2013/14			2014/15	
	Estimate	SE	Estimate	SE	Estimate	SE	Estimate		SE	Estimate	SE
Pre‐operative criteria—common to all years											
Pre‐criteria failed	−0.177***	0.068	−0.109**	0.051	−0.143***	0.048	−0.101**	0.045		−0.105***	0.021
Pre‐criteria failed × England	0.068	0.069	0.014	0.053	0.060	0.049	0.033	0.046		0.052**	0.022
Pre‐operative criteria including BPT4 (introduced in 2012)											
Pre‐criteria failed					−0.149***	0.046	−0.102**	0.046		−0.105***	0.020
Pre‐criteria failed × England					0.067	0.048	0.034	0.046		0.051**	0.022
Hospital fixed effects	X		X		X		X			X	
Time (month) fixed effects	X		X		X		X			X	
Patient characteristics	X		X		X		X			X	
Number of hospitals	178		179		178		178			176	
Number of observations	36,332		46,090		51,277		55,680			59,064	

*Note:* In this cross‐sectional specification, the independent effect of being treated in England is subsumed into the hospital fixed effect. Standard errors are clustered at hospital level.

*** *p* < 0.01.

** *p* < 0.05.

* *p* < 0.1.

**TABLE A5:** Correlations between pre and post policy hospital level of BPT achievement—England.

	BPT1	BPT2	BPT3	BPT4	BPT6	BPT7	BPT8
Pre‐policy							
BPT2	0.0533471						
BPT3	0.0852617	0.4540524					
BPT4	0.1757829	0.161227	0.046969				
BPT6	0.021778	0.4273587	0.3258187	0.0382021			
BPT7	0.1376978	0.3160341	0.7986739	−0.0175725	0.4644414		
BPT8	0.1398047	0.0866402	0.0869926	0.1678116	0.0720509	0.1277451	
BPT9	0.1889081	0.0422909	0.0633095	0.3464491	0.1422032	0.0477978	0.490464
Post‐policy							
BPT2	0.1718149						
BPT3	0.2569049	0.7444319					
BPT4	0.2531866	0.3104307	0.3800543				
BPT6	0.3299375	0.7745612	0.6588883	0.3535784			
BPT7	0.1494381	0.6734034	0.489723	0.2060501	0.6189031		
BPT8	0.1818794	0.586477	0.4159776	0.2293943	0.570452	0.7366091	
BPT9	0.1977822	0.6372785	0.5964103	0.3754912	0.6913331	0.6448492	0.5107453

**TABLE A6:** Correlations between pre and post policy hospital level of BPT achievement—Wales.

	BPT1	BPT2	BPT3	BPT4	BPT6	BPT7	BPT8
Pre‐policy							
BPT2	0.4410099						
BPT3	−0.7259837	−0.0963503					
BPT4	0.692365	0.5175061	−0.7229286				
BPT6	0.2332274	−0.0345799	−0.2685128	−0.1542094			
BPT7	−0.2472761	−0.2504135	0.158914	−0.6293151	0.5332416		
BPT8	0.776419	0.599652	−0.3829879	0.444914	0.4837796	0.0585489	
BPT9	−0.0291473	−0.2032991	−0.4230165	0.292743	0.4156475	−0.0366301	−0.0928712
Post‐policy							
BPT2	−0.1106864						
BPT3	−0.1254255	0.0177983					
BPT4	0.430889	0.3316164	0.1290888				
BPT6	0.5380011	0.0239367	0.0712691	0.4063503			
BPT7	0.1852012	0.0955939	0.3835205	−0.354855	0.3032882		
BPT8	0.0205934	0.1529692	0.1363469	0.0689489	0.6224649	0.2598522	
BPT9	−0.091108	−0.5386822	0.0760156	−0.2151017	0.1057863	0.1654165	0.1527502
